# anNET: a tool for network-embedded thermodynamic analysis of quantitative metabolome data

**DOI:** 10.1186/1471-2105-9-199

**Published:** 2008-04-16

**Authors:** Nicola Zamboni, Anne Kümmel, Matthias Heinemann

**Affiliations:** 1 ETH Zurich, Institute of Molecular Systems Biology, Wolfgang-Pauli Strasse 16, 8093 Zurich, Switzerland

## Abstract

**Background:**

Compared to other omics techniques, quantitative metabolomics is still at its infancy. Complex sample preparation and analytical procedures render exact quantification extremely difficult. Furthermore, not only the actual measurement but also the subsequent interpretation of quantitative metabolome data to obtain mechanistic insights is still lacking behind the current expectations. Recently, the method of network-embedded thermodynamic (NET) analysis was introduced to address some of these open issues. Building upon principles of thermodynamics, this method allows for a quality check of measured metabolite concentrations and enables to spot metabolic reactions where active regulation potentially controls metabolic flux. So far, however, widespread application of NET analysis in metabolomics labs was hindered by the absence of suitable software.

**Results:**

We have developed in Matlab a generalized software called 'anNET' that affords a user-friendly implementation of the NET analysis algorithm. anNET supports the analysis of any metabolic network for which a stoichiometric model can be compiled. The model size can span from a single reaction to a complete genome-wide network reconstruction including compartments. anNET can (i) test quantitative data sets for thermodynamic consistency, (ii) predict metabolite concentrations beyond the actually measured data, (iii) identify putative sites of active regulation in the metabolic reaction network, and (iv) help in localizing errors in data sets that were found to be thermodynamically infeasible. We demonstrate the application of anNET with three published *Escherichia coli *metabolome data sets.

**Conclusion:**

Our user-friendly and generalized implementation of the NET analysis method in the software anNET allows users to rapidly integrate quantitative metabolome data obtained from virtually any organism. We envision that use of anNET in labs working on quantitative metabolomics will provide the systems biology and metabolic engineering communities with a mean to proof the quality of metabolome data sets and with all further benefits of the NET analysis approach.

## Background

Metabolomics, the technique to measure intra- and extracellular small molecules, was introduced a few years ago as the youngest child in the omics family. The technique provides data that, for example, can help us to complement our picture of metabolic pathways through identification of novel metabolites, or – by means of statistical analyses – to spot metabolic differences between strains or conditions [1,2].

Beyond these already valuable qualitative insights, however, further interpretation of metabolite data is difficult. This is due to the fact that the metabolome does not have a direct link to the genome such as mRNA or proteins. Furthermore, metabolite concentrations are the result of a multitude of interrelated molecular actions ranging from the gene expression level to the metabolic level and consequently the cause for an increased or decreased metabolite concentration is not intuitively accessible. Hence, in order to obtain mechanistic biological insights from metabolome data, we need rigorous integration in mathematical models [3,4]. Here, an obvious strategy would be the integration of metabolome data in kinetic models [5,6]. To date, however, this approach is still impracticable because of the sparse knowledge about *in vivo *reaction mechanisms and kinetic parameters. In addition, the continuing challenges in the area of computational analysis [7] make it very unlikely that large-scale kinetic models will be available in the near future. In brief, there is a pressing need for computational methods that allow extracting mechanistic insights from quantitative metabolome data.

Apart from the lack of suitable methods for interpretation of metabolome data, also the quantitative measurement of intracellular metabolite concentrations still faces serious issues [8,9]. Specifically, experimental problems arise from the technical difficulty of sampling rapidly enough to avoid artifacts for metabolites with fast turnover rate, and from the heterogeneous nature of the species that compose the metabolome, which calls for complex and thus error prone sample preparation procedures and diversified analytical platforms. Due to the numerous potential pitfalls associated with concentration measurements, a computational method that can check supposedly quantitative dataset for potential errors is highly desired to guarantee high-quality data to be used in further analyses, such as in computational systems biology [10].

Recently, we presented a method called network-embedded thermodynamic (NET) analysis that can be utilized to address both aspects [11]: NET analysis can check for thermodynamic inconsistencies in quantitative metabolome data sets and can extract mechanistic biological insights from these data. In brief, NET analysis couples metabolite concentrations to an operating metabolic network via the second law of thermodynamics and the metabolites' Gibbs energies of formation. The underlying optimization framework determines the feasible range (i.e. upper and lower bounds) of the Gibbs energy of a particular reaction *k*, Δ_*r*_*G*'_*k*_,, using metabolite concentrations *c*_*i*_, reaction directionalities *r*_*j*_, the reaction stoichiometry of a metabolic network *s*_*ij *_and predetermined standard formation energies Δ_*f*_*G'*°_*i*_:

(1) $$\begin{array}{l} \min/\max\Delta_{r}G{'}_{k}\\ \begin{array}{ll} \text{subject to} & \begin{array}{l} -\Delta_{r}G{'}_{j}\cdot sign(r_{j})<0\\ \Delta_{r}G{'}_{j}=\sum_{i}s_{ij}\Delta_{f}G{'}_{i}\\ \Delta_{f}G{'}_{i}=\Delta_{f}G'{{}^{\circ}}_{i}+RT\ln(c_{i})\\ c_{\min}\leq c_{i}\leq c_{\max} \end{array} \end{array} \end{array}$$

It should be emphasized that the reaction directionalities *r*_*j *_are not equivalent to enzyme reversibility. A non-zero reaction direction *r*_*j *_implies a non-zero net flux of reaction *j*. Lower and upper bounds for each reaction's Gibbs energy are determined by minimization and maximization of Δ_*r*_*G'*_*k *_in the non-linear system described Eq. (1), respectively. NET analysis can indicate, in which direction a reversible enzyme is operating under the experimental conditions: When the estimated upper bound of Δ_*r*_*G'*_*k *_is negative, the net flux of reaction *k *can only proceed in the forward direction. Analogously, when the estimated lower bound of Δ_*r*_*G'*_*k *_is positive, the net flux of reaction *k *can only proceed in the reverse direction.

In addition, a determined displacement of Δ_*r*_*G'*_*k *_from zero reflects the distance, at which the particular reaction operates from equilibrium. Reactions that operate far from equilibrium (at least 10 kJ mol^-1^) are likely to be actively regulated. No conclusions can be drawn if lower and upper bounds are negative and positive, respectively, as estimated on the basis of the provided measurement data and constraints. Similarly, also the feasible ranges for metabolite concentrations *c*_*j *_can be determined by optimization. For detailed information on the method, the reader is referred to [11].

So far, the routine for network-embedded thermodynamic analysis was only available as a research tool that was not appropriate for non-expert users. In order to facilitate a more widespread use, we developed a Matlab-based software called anNET. anNET is a generalized implementation of NET analysis and thus can be applied (i) for consistency analysis of measured metabolite concentrations, (ii) for prediction of metabolite concentrations beyond the actually taken measurements and (iii) for identification of putative active sites of genetic or allosteric regulation. anNET is generalized to the extent that virtually any cellular reaction network (including compartmentalized reaction networks) and any set of quantitative metabolome data can be integrated. Its user-friendly implementation does not sacrifice correctness of the analysis even in complex cases. The precondition is a stoichiometric model that describes the metabolic network of interest. Notably, the size of the model can span from one reaction to a comprehensive genome-wide reconstruction of metabolism. In large to genome-wide models, the resolving power of a NET analysis increases only when metabolome data or flux directions are available on peripheral pathways. Also when applied to a single reaction, NET analysis can verify whether measured concentrations are compatible with the expected flux direction.

It is important to note that unknown pathways missing in the used metabolic network model as well as unknown and thus not specified flux directions, or unavailable thermodynamic data for certain metabolites will never render a data set infeasible, and thus NET analysis is rather conservative. Further, thermodynamic feasibility is only a necessary but not a sufficient condition for correct quantification of metabolite concentrations. However, NET analysis as an easy-to-apply tool can test for major experimental errors, while relying only on indisputable (i.e. thermodynamic) facts.

We envisage that anNET will be used in labs working on quantitative metabolomics to check for thermodynamic consistency of metabolome data and, thus, pinpoint compound-specific flaws in the analysis procedure from sampling to quantification. We hope this will contribute to further improve the quality of metabolome measurements.

In the following, we give a brief overview on possible utilizations of anNET, and then describe the algorithmic workflow, the user interface, the details of how cellular compartments are handled as well as depict two additional tools that support NET analysis to (i) identify errors in thermodynamically infeasible data sets and to (ii) identify minimal flux direction sets in metabolic reaction networks. Then, we demonstrate the validity of the generalized NET analysis implementation in anNET and compare different solvers, we apply anNET to recently published metabolome data sets and demonstrate its novel function to troubleshoot infeasible data sets.

### Applications

Examples for applications of anNET are listed in Table 1. A NET analysis has three inputs: a stoichiometric model, metabolite concentrations, and flux directions. Technically only the first is compulsory, but in practice all available information is typically used to constrain the optimization and yield the most detailed results. Depending on the goal of the analysis (Tab. 1), the user can choose to estimate either the feasible ranges of concentrations for every metabolite in the model with known Δ_*f*_G°, feasible ranges of Δ_*r*_G' for each reaction, or values for arbitrary non-linear terms such as the oxidation state of NADH/NAD^+ ^or the energy charge of the adenylate pool.

**Table 1:** Examples of anNET applications

**Application**	**Ranges estimated**	**Notes**
Check thermodynamic consistency	none	Check only once feasibility. For non-feasible systems, use the troubleshooting routine.
Estimate unmeasured concentrations	concentrations	
Resolve concentrations in different compartments	concentrations	
Find minimum/maximum feasible NADH/NAD ratio or adenylate energy charge	Non-linear terms	Define the ratio of interest as input with very loose (wide) bounds
Infer reaction direction	Δ_*r*_G'	Find reactions that have a Δ_*r*_G' with a unique sign
Verify reversibility in model	Δ_*r*_G'	Infer reaction directions from NET analysis and compare with reversibility in model or literature
Spot putative control sites	Δ_*r*_G'	Find reactions that are known to be active and operate far from equilibrium
Exclude activity of transporters	Δ_*r*_G'	Transporters with non-zero, positive Δ_*r*_G' are either not active or have reversed flux.

## Implementation

### Workflow

**Figure 1:**
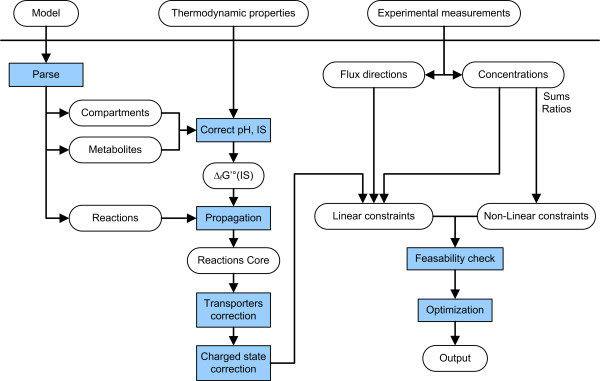
Analysis workflow in anNET.

The general workflow of anNET is depicted in Figure 1. All input data are parsed from spreadsheets every time an analysis is requested. The available thermodynamic data and the specified pH and ionic strength values are then used to calculate the transformed standard Gibbs energy of formation, Δ_*f*_G'°, for all metabolites and pseudoisomers in each compartment. From the complete list of reactions in the model, only those for which all reactants have a known Δ_*f*_G'° are selected to constitute the core model that is used for NET analysis. From this set of reactions, a system of linear constraints is built that describes Eq. (1). A set of routines is then called to handle the special cases of transporters (described below) and of reactions that only convert reactants in a specific charge state. The overall standard Gibbs formation energies of reactants that can occur in two or more charge states are calculated by combining the standard Gibbs formation energies of the pseudoisomer according to their molar fractions.

Next, the provided information on known flux directions is added as linear constraints to constrain the respective reactions' Gibbs energies, Δ_*r*_G'. The provided metabolite data is handled according to whether the measurement specifies a concentration of a single intermediate, a sum of multiple concentrations, or a ratio of concentrations. Concentration data that relates to a single intermediate in a specific compartment directly translates into lower and upper bounds for that concentration. As a consequence, these kinds of constraints preserve linearity in Eq. 1 because we take the logarithm of the concentrations as variables in the optimization. In contrast, the provision of concentration sums and ratios result in non-linear constraints.

A first single optimization is performed to check whether the defined system is actually feasible, meaning that it is not contradictingly constrained by either flux directions or measured concentrations. If the system happens to be infeasible, a special routine is called to spot the conflicts in the dataset (see below). A successful feasibility test implies that the measured data is thermodynamically consistent. In this case, a complete NET analysis is performed by cycles of minimization and maximization that determine the feasible ranges of Gibbs reaction energies, of metabolite concentrations and of the non-linear terms that the user had specified to be estimated.

### Interface

**Figure 2:**
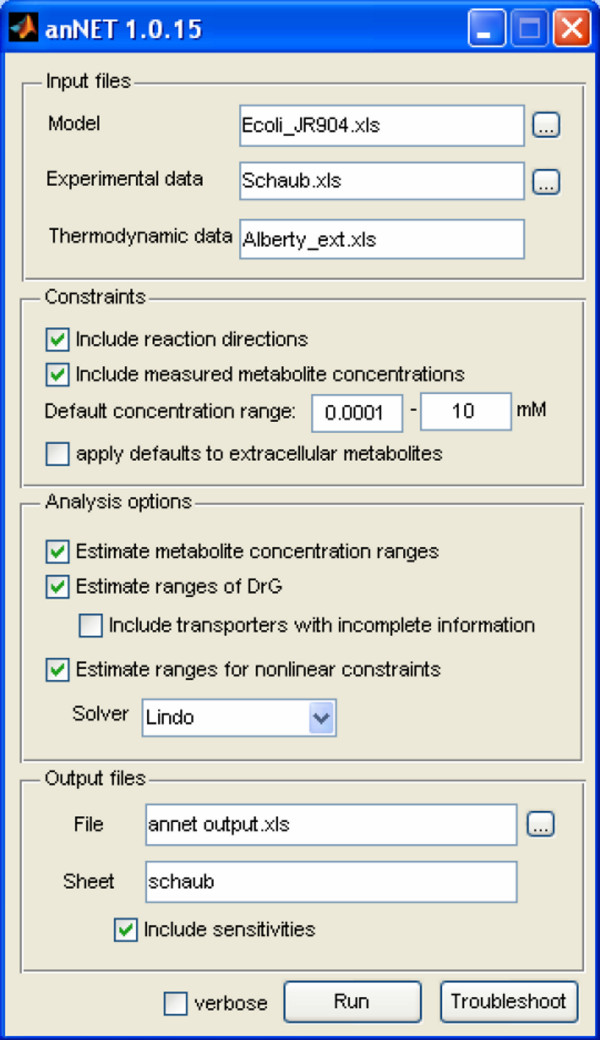
The graphical user interface permits to submit all parameters and options that are necessary to accomplish a NET analysis.

A single main window (Figure 2) acts as graphical user interface to pass all inputs and options to the core routines. The dialog is divided into four panels, which are connected to the modules that are executed in sequence each time the analysis is invoked: definition of input files, definition of constraints, choosing options for the analysis and definition of the output files.

In the first panel, the user specifies where the information required to execute a NET analysis can be retrieved. Inputs are grouped into three categories: (i) defining the stoichiometric model and physicochemical properties of compartments (i.e. pH and ionic strength), (ii) specifying metabolite concentrations and flux directions, and (iii) providing the thermodynamic input data (i.e. the standard formation energies). All these input data are provided via flat files (comma separated values) or Excel spreadsheets using an intuitive syntax.

More specifically, the stoichiometric model is entered as an array of reactions. Each reaction is defined with reactants of unique names, the stoichiometric coefficients, and the compartment, in which the reaction takes place. Transport processes between cellular compartments or between the intra- and extracellular environment are defined analogously and are automatically recognized as transport processes, when two or more compartments are defined for the participating reactants. For cases, where a participating reactant can only be transported in a specific charge state (e.g. some transport reactions can only shuttle either neutral or charged species), the user can also define reactants' charge states in order to perform a mechanistically more correct NET analysis. Based on the provided list of reactions, a list with all metabolites occurring in the stoichiometric network is generated by anNET.

Measured metabolite concentrations can be entered by the user as exact values, as ranges, as sum of concentrations of two or more metabolites, or as ratios between (sums of) concentrations of multiple metabolites. Hence, anNET seamlessly can process semi-quantitative metabolome data (i.e. ratios of metabolite concentrations) and pooled concentrations of e.g. structural isomers that could not be resolved by the employed separation or analytical technique. Known flux directions are defined in a separate worksheet with the flux sign. Thermodynamic properties are provided in a list that for each metabolite specifies the standard Gibbs energy of formation (if known), its charge, and the number of hydrogen atoms. These three quantities enable to accurately estimate the Gibbs energy of formation as a function of pH and ionic strength – specific for each compartment [12]. Notably, for reactants that at physiological pH can occur in more than one charge state (so-called pseudoisomers, e.g. all amines, organic acids, or phosphorylated compounds), the mentioned three quantities are reported for each one of them. The relative abundance of the different pseudoisomers in the different compartments and thus the overall charge state of an intermediate is then calculated automatically by anNET. Currently, with anNET we enclose thermodynamic properties for more than 200 metabolites (more than 350 pseudoisomers) that condense a decade of experimental values published by Robert A. Alberty [13] and which we further extended and curated. Since this set of thermodynamic data covers most of the analytes that can be detected in routine metabolome experiments, we decided not to include Gibbs energies of formation that were determined via the group contribution approach [14] because they partly significantly deviate from experimentally determined values. Nevertheless, the provided list of the Gibbs energies of formation can be freely extended or be replaced by the user via the respective spreadsheet.

The second panel of the graphical user interface affords a mean to rapidly set the constraints for the analysis. In the third panel, the user can decide what has to be estimated and which solver to use (if multiple are available), while in the fourth panel different options for reporting and the destination of the reported result can be defined. Currently, anNET can utilize two optimizers to solve the nonlinear optimization: *fmincon *from the Matlab Optimization Toolbox (The Mathworks) or the LINDO API library (LINDO Systems Inc.). The computational performance of the two solvers is compared in the Result section.

### Handling of compartments and transporter reactions

To cope with biochemical reaction networks of virtually any organism, anNET has the capability to handle compartmentalized models and various kinds of transport reactions. For this purpose, metabolites present in different compartments are treated as independent entities and compartment-specific Gibbs energies of formation are computed (see also above). By default, the pools of a metabolite that is present in different compartments cannot exchange unless a respective transport process is defined in the model. Most metabolome platforms do not allow distinguishing between compartments and the measurements reflect the average over the entire cell. Therefore, a measured concentration typically translates into a constraint on the sum of the concentrations in all compartments weighted by the compartment volume. The context of the network operation that is specified by the user in terms of flux directions provides – amongst other things – also information about active transport processes across compartmental boundaries. These transport processes together with the network operation as a whole in several cases then enforce a distinct distribution of metabolite concentration in the different compartments. For example, if a metabolite has to cross a membrane separating two compartments by passive diffusion, then a fall in concentration must exist at steady state to sustain the flux.

**Figure 3:**
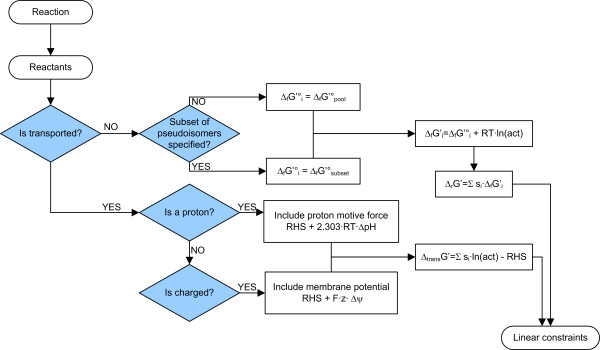
Extensions of linear constraints to integrate the thermodynamics of transport processes and charge-specific catalysis.

Two aspects must be taken into account to calculate the Δ_*r*_G' of a transport process: First, proton transport is affected by the pH gradient between the two compartments. Second, when a charged molecule is transferred across a membrane with an electrical potential, the thermodynamics of acting upward or downward the membrane potential has to be considered. anNET automatically checks and corrects for these potential contributions (Figure 3). In our implementation, these steps are merged with the correction for pseudoisomers and do not increment the size of the optimization model.

### Identifying potential errors in infeasible data sets

Infeasible systems occur when constraints on concentrations and/or flux directions are conflicting such that no solution exists. Only in very rare cases, we observed that infeasibility was an artifact of the non-linear solver, which failed to spot a feasible solution e.g. because of numerical, scaling, or convexity issues.

Tracking the source of infeasibility in the non-linear problem of the NET analysis is problematic as in most cases only the combination of several constraints causes an infeasibility and thus pinpointing a unique source for an infeasibility is not possible. For this reason, we opted for a practical semi-combinatorial approach.

The developed approach seeks to identify conflicts between pairs of metabolite concentrations or between metabolite concentrations and flux directions. Here, we developed a two stage procedure: In the first stage, all defined constraints on reaction directions are set to be active, whereas all bounds on metabolite concentrations are first removed from the system. Then, upper and lower bounds on concentrations are introduced sequentially – one metabolite at the time. The sequence of additions is ordered such that first the metabolites are reintroduced, for which no measurement value was available (i.e. for which broad default concentration bounds were defined), followed by metabolites, for which measurement values were specified in the input spreadsheet, and then followed by the non-linear expressions for concentration sums and ratios.

Whenever inclusion of a metabolite leads to an infeasible system, the metabolite is blacklisted and the corresponding constraint is removed again. Once all concentration constraints and the non-linear constraints are tested, the blacklisted metabolites are taken to the second stage, where the flux direction constraints conflicting with the blacklisted metabolites are screened by a similar combinatorial approach. Overall, this procedure delivers a list of conflicting pairs of concentration/concentration constraints or of concentration/reaction direction constraints, which are useful in spotlighting infeasible subsystems.

### Prediction of the minimum flux directions set

Generally, the amount of information gathered from a (correctly quantified) metabolite data set scales with the number of constraints imposed on the system [11]. As the provision of flux directions heavily constrains the system, it is desirable to define as many flux directions as possible. Such definition, however, is somewhat problematic for peripheral pathways, for which typically no information can be obtained from experimental ^13^C-flux analysis [15]. For this reason, we devised a computational tool that predicts the minimum flux direction set from a metabolic network model on a given substrate.

The underlying idea is the following: Growing cells must synthesize certain biomass components. These components must either be taken up from the extracellular medium or they must be synthesized from the nutrients. Provided a certain medium composition, this fact can be used to predict a set of reactions whose flux must be non-zero and must be oriented in a certain direction to ensure that all biomass precursors can be synthesized. The only requisite for this analysis is a list of biomass components. Curated biomass models exist for most of the available manually reconstructed genome-scale metabolic network models [16,17]. In the case of poorly described organisms, only validated precursors should be included. Omission of an essential intermediate from the list of biomass components does never restrict flux variability and, thus, it does not invalidate the minimal flux direction set obtained when starting from an incomplete list. A routine to scavenge this minimum set of flux directions is distributed with anNET. The algorithm is based on flux balance analysis [18] and minimizes and maximizes the flux through each reaction in the stoichiometric model under the constraint that the biomass yield is non-zero. For this analysis presented here, we omit all reaction direction definitions that typically come along with these models and rather use a fully reversible model in order to reduce the number of false positives that could be obtained in case of incorrectly defined reaction irreversibilities in these models. Further, here, we allowed all metabolites that we not explicitly declared as substrates to be produced if this is necessary for biomass formation. It should be noted that the user can freely modify these assumptions and that also models with defined reaction directions (for example derived from systematic assignment [19]) can be employed for this analysis.

## Results and discussion

### Validation of the implementation

To ascertain the correct NET analysis implementation in anNET, we analyzed the *E. coli *dataset published by Schaub *et al. *[20] with anNET using the iJR904 model [21] (see Additional file 1). The results obtained with anNET were compared to the published results that were obtained independently with the NET analysis implementation based on the non-generalized code [11]. The original model of 923 reactions and 762 metabolites was reduced to a core model with 166 reactions and 147 metabolites after the available thermodynamic information was propagated. The data set from Schaub *et al. *consisted of 6 metabolite concentrations and 4 sums of concentrations that resulted from not fully analytically resolved analytes. Further 3 ratios were added to the system to assess the feasible range of the adenylate energy charge (AEC) and the redox state of the cofactors NAD^+^/NADH and NADP^+^/NADPH. The input concentration ranges of these three ratios were chosen very wide to avoid that they become active constraints. Notably, the two analyses delivered equivalent results for all ranges of concentrations and of Δ_*r*_G (see Additional file 2). Minor variations are caused by the fact that in the previously published NET analysis an uncertainty for all Δ_*f*_G° of ± 0.5 kJ/mol was employed to account for possible errors in the thermodynamic input variables.

### Comparison between solvers

Two different non-linear solvers can be used by anNET for the optimization, i.e. the LINDO API library, which relies on the CONOPT3 algorithm, or the *fmincon *function from the Matlab Optimization Toolbox. Independent NET analyses of the aforementioned *E. coli *dataset with the two solvers delivered identical results for metabolite concentration and Δ_*r*_G estimates (see Additional file 3), thus validating the robustness of the solution. However, it should be noted that *fmincon *occasionally failed to minimize/maximize the value of concentration ratios. For example, in the data set from Schaub *et al.*, the ranges for the summation constraints and the adenylate energy charge (i.e. resembling a ratio) were estimated in agreement with LINDO, while *fmincon *underestimated the feasible ratios between NADP/NADPH and NAD/NADH. Despite several modifications in the optimization settings, including the starting point and maximum duration, we were not able to find a universal configuration that lead to robust optimization of non-linear terms with *fmincon*. Furthermore, the LINDO solver consistently proved to complete the optimization 2–3 orders of magnitude faster than *fmincon *(Table 2). The speed of the solvers did not significantly improve when explicit functions to calculate the gradients of the non-linear terms or the objective function were provided. The computation time of the *fmincon *solver could be decreased by almost one order of magnitude by allowing less restrictive optimization tolerance criteria. Unfortunately, this resulted occasionally in premature termination and thus sub-optimal results. For reasons of robustness and speed, we opted to utilize the LINDO library for all following analyses.

**Table 2:** Comparison of performance of *fmincon *and LINDO solver for estimation of feasible ranges.

		Solver	
Computation time for	Ranges to estimate	*fmincon*	LINDO
- parsing		20 ± 1 s	20 ± 1 s
- feasibility check	1	25 ± 3 s	0.2 ± 0.1 s
- ranges of concentrations	166	51 min	23 s
- ranges of ΔrG	147	145 ± 20 min	30 s
- non-linear constraints	7	n.d.^a^	1 s

The time is given for at least duplicate analyses of the Schaub data set on a Pentium IV 3 GHz processor. Note: ^a^, no runtime is provided because no robust optimization was possible (see text).

### Application of anNET to published metabolome data sets

We tested the thermodynamic consistency of three recently published metabolome data by Schaub *et al. *[20], Hiller *et al. *[22], and Ishii *et al. *[23], all of which relate to wild-type *E. coli *glucose-limited continuous cultivations at a growth rate of 0.10–0.13 h^-1^. For these conditions, fluxes in central carbon metabolism were measured experimentally by ^13^C metabolic flux analysis [24,25]. We used this information to manually compile a list of 36 direction constraints in central carbon metabolism (which, in the following, we refer to as 'Set 1'). An independent second set of direction constraints was obtained *in silico *using our above mentioned tool for the prediction of the minimum set of essential flux directions. For growth on glucose and by using the biomass vector specified in the model iJR904 [21], we obtained a total of 131 reactions ('Set 2') that need to be active under the assumption that all reactions in the model are reversible. Notably, all these reactions are located in peripheral regions of the metabolism, where unique biosynthetic routes to the biomass precursors have to be active. A knockout in these genes is lethal unless the model topology or the biomass vector is ill-defined. Interestingly, by this approach no flux direction is predicted in central carbon metabolism, where multiple alternative pathways exist. Owing to the complementary nature of Set 1 and Set 2, we merged them to construct Set 3.

We found that not all of the three data sets were thermodynamically feasible, even when we allowed a 10% error on all measured concentrations (Table 3 and Additional file 4). Consistent with the previous analysis [11], the Schaub data set was proven to be feasible with all sets of flux constraints. In contrast, both the Hiller and the Ishii data sets were not feasible when the set of flux constraints obtained from ^13^C flux analysis was employed.

**Table 3:** Consistency check of three recent *E. coli *metabolome datasets. [Supplementary-material S4]

	Measured concentrations				Constraints on flux directions		
Data set	CCM	Redox cofactors	Energy carriers	Others	Set 1	Set 2	Set 3 (= Set 1 + Set 2)
Schaub	8	0	2	0	F	F	F
Hiller	8	3	3	1	NF	F	NF
Ishii	14	5	3	71	NF	F	NF

The flux directions sets are described in the main text. Abbreviations: F, feasible; NF, not feasible, CCM, central carbon metabolism. Details can be found in Additional file 4

### Troubleshooting of non-feasible systems

We used our troubleshooting routine to localize the conflicts that provoke the infeasibility in the above datasets. Despite the large number of measured metabolites in the dataset of Ishii *et al. *and the therewith involved increased risk for system infeasibility, only one apparent thermodynamic inconsistency was found to exist in the data set, which is the concentration range of ribulose-5-phosphate (ru5p-D) (see Additional file 5). Conflicts were found to exist with the concentration of ribose-5-phosphate (r5p) and the directions of three enzymes: ME2, ICDHyr, and RPI. Removal of the directions constraints for ME2 and ICDHyr did not relax the unfeasibility, thus locating the inconsistency around RPI, which catalyzes the isomerization between ru5p-D and r5p. In fact, removal of the measurement of ru5p-D or r5p, or of the RPI reaction direction constraint turned the system into a feasible system. Owing to the high confidence of the RPI flux direction estimate based on ^13^C metabolic flux analysis, we conclude that the problem is likely due to an erroneous concentration. From thermodynamics, roughly equimolar concentrations are expected for the two intermediates ru5p-D and r5p, whereas a 4–5 fold higher amount was detected for ru5p-D. Interestingly, Ishii *et al. *reported additional wild-type metabolome data sets for different growth rates: four out of five wild-type data sets exhibited the same inconsistency.

In the data set by Hiller *et al.*, our analysis identified two problematic concentration ranges: glucose-6-phosphate (g6p) and glyceraldehyde-3-phosphate (g3p) (see Additional file 6). In the first case, measured g6p concentrations are not compatible with the assumed direction of the phosphoglucoisomerase (pgi). In glucose-limited continuous cultures, the glycolytic flux through the pgi is directed from g6p to fructose-6-phosphate (f6p) [24]. Because of the resulting constraint on Δ_*r*_G'(pgi), the concentration of g6p has to be at least 3.1-fold larger than that of f6p, in contrast with the measured ratio of 2.3. The conflict is relieved when a relative error of at least 25% is allowed for both concentrations. In the second problem, g3p is incompatible with the concentrations of dihydroxyacetone-phosphate (dhap) and fructose-1,6-bisphosphate (fdp) and the connecting reactions catalyzed by the triosephosphate isomerase (tpi) and the fdp-aldolase (fba). The reaction directions imposed by the glycolytic flux dictate that the g3p concentration has to be in the range between 2–38 μM when the concentrations of fdp and dhap are assumed to be within 30% of the measured values. This range, however, is largely lower than the measured g3p value of 200 μM. Interestingly, no feasible system could be obtained when removing the experimental concentrations of either fdp or dhap from the dataset, because this resolved the infeasibility around either fba and tpi, respectively, but not both simultaneously. Overall, these examples demonstrate the usefulness of the troubleshooting function to identify the loci of thermodynamic infeasibility and to suggest potential error sources.

In general terms, it is important to emphasize two aspects. Firstly, apparent inconsistencies in metabolite concentrations may be linked to bad measurements but also reflect faulty thermodynamic data or local differences in reactant activity. The troubleshooting routines can not distinguish between these causes, but diagnoses all of them simultaneously by the requisite to further relax concentration constraints around specific nodes. Secondly, the fact that modification or removal of one constraint (or more) in an unfeasible system lead to a feasible one proves neither that the modified constraints were wrong, nor that the others were correct. It is a mere indication that requires experimental verification.

## Conclusion

anNET is the first tool publicly available for network-embedded thermodynamic analysis of metabolome data. The most immediate application of anNET is the consistency check of quantitative metabolome measurements [11]. As outlined in several recent papers [8,26], reliable quantification of intracellular metabolites is still extremely challenging. Thus, anNET can help here.

In this context, however, it is important to note that thermodynamic feasibility approved by NET analysis is not a sufficient condition to certify that the measured concentrations reflect the true state of a cell. Nevertheless, despite the rather conservative quality filter that is given by NET analysis, a previous study showed that out of seven published metabolite datasets, three were thermodynamically not consistent [11]. A data set that fails to be thermodynamically consistent must be carefully checked before it is used for further analyses that rely on quantitative information. To this respect it is important to stress that in an unfeasible system not only the experimental data should be questioned, but also the respective input data (i.e. assumed reaction directions, thermodynamic data) as well as the inherently underlying assumptions (i.e. well-mixed compartments).

The prerequisites for a consistency check by NET analysis is that (i) quantitative metabolomics data is available (although relative amounts in form of concentration ratios can also be integrated by anNET); and (ii) flux directions can be defined. Hence, this precludes the application of NET analysis to the consistency check of for example serum metabolome, or to cells grown in rich media were flux directions are uncertain. We hope that anNET will soon be used for quality check of quantitative metabolome data and thus, in consequence, the quality of published quantitative metabolite data sets will rise.

## Availability and requirements

Project name: anNET

Operating system: tested on Microsoft Windows XP and Linux Red Hat.

Programming language: tested with Matlab 7.0 and later (The Mathworks).

Other requirements: Matlab Optimization Toolbox (The Mathworks) or LINDO API, versions 2.0 – 5.0 (LINDO Systems Inc.)

License: freely available from the authors for academic purposes.

Any restriction to use by non-academics: license required.

## Authors' contributions

AK developed initial prototypes. NZ generalized and optimized the software. All authors participated to validation. NZ and MH wrote the manuscript. All authors read and approved the final manuscript.

## Supplementary Material

Additional file 1 Metabolic model used for *E. coli*. Click here for file

Additional file 2 Comprehensive anNET analysis of the data set by Schaub. Click here for file

Additional file 3 Comparison of LINDO and *fmincon *results (Supporting material to Table 2) Click here for file

Additional file 4 Detailed anNET results for analysis of the three data sets reported in Table 3. Click here for file

Additional file 5 Troubleshooting of Ishii data set. Click here for file

Additional file 6 Troubleshooting of Hiller data set. Click here for file
